# The Use of Amantadine in Treating Extrapyramidal Symptoms in Organophosphates Poisoning in a Child

**DOI:** 10.1155/2023/1632052

**Published:** 2023-09-04

**Authors:** Wesam Althaqafi, Reham Ibrahim Alanazi, Wadha Khalid Almeshari

**Affiliations:** ^1^King Abdullah Specialized Children's Hospital (KASCH), King Abdulaziz Medical City, Riyadh, Saudi Arabia; ^2^King Salman Abdulaziz Medical City, Ministry of Health, Madinah, Saudi Arabia; ^3^King Saud Medical City, Ministry of Health, Riyadh, Saudi Arabia

## Abstract

*Background*. Organophosphates are chemicals that lead to the accumulation of acetylcholine, causing muscarinic symptoms such as salivation and nicotinic manifestations like muscle weakness and hypertension and rarely leading to basal ganglia impairment, manifesting as extrapyramidal symptoms. Literature reported that the use of amantadine, an amine that has both antiviral and dopaminergic activities, improves extrapyramidal manifestations. Most of the studies exploring the effect of amantadine were conducted on adults and there are extremely limited data in regards to this topic in Saudi Arabia. Thus, the purpose of this case study is to report the outcome of treating a child who developed extrapyramidal symptoms due to organophosphates poisoning with amantadine. *Case Description*. A 6-year-old boy was found by his family drowsy and drooling with an insecticide bottle beside him. He was brought to the ER and arrested on arrival, and he was eventually revived after 5 minutes of CPR. Then, he developed features of extrapyramidal involvement such as delirium, hyperactivity, akathisia, aphonia, and tremors. He was started on oral amantadine 50 mg once daily and then increased to twice daily for two weeks while admitted. During admission, his symptoms were assessed daily, and an improvement was noticed by his family and the medical team. Upon discharge, he was able to form sentences; tremors were almost resolved; and there was no rigidity or agitation. He was followed up postdischarge and showed significant improvement. He continued amantadine for almost 3 months until the full resolution of his symptoms. *Discussion and Conclusion*. This case illustrates the promising benefits of using amantadine in treating extrapyramidal manifestations following organophosphate ingestion.

## 1. Introduction

Organophosphate compounds are a group of chemicals that were first synthesized in the early 1800s and are used in both industrial and domestic settings [1]. Worldwide, approximately 3,000,000 people per year experience organophosphates poisoning [2]. Organophosphates inhibit acetylcholinesterase (AChE) which is responsible for the breakdown of acetylcholine, thus leading to its accumulation and stimulation of cholinergic receptors [3].

Organophosphates poisoning is a clinical diagnosis [1]. The aforementioned mechanism leads to a state known as a cholinergic syndrome, characterized by muscarinic symptoms such as salivation, lacrimation, urination, vomiting, and meiosis, with nicotinic manifestations like muscle weakness, fasciculation, diaphragmatic failure, hypertension, and tachycardia [4]. In rare conditions, exposure to these neurotoxic organophosphates can lead to basal ganglia impairment and parkinsonism, which develops postrecovery from the acute cholinergic crisis, and can be seen as rigidity, bradykinesia, or choreoathetosis [5].

Organophosphates poisoning is treated with the injection of atropine, an anticholinergic drug, along with pralidoxime to reactivate acetylcholinesterase [4, 6]. Moreover, anticonvulsants are usually added to the regimen to prevent seizures during the cholinergic crisis [4]. The literature reported that the use of amantadine, an amine that has both antiviral and dopaminergic activities, improves parkinsonism following organophosphates poisoning [7, 8].

Initially, amantadine was discovered to have inhibitory effects against several strains of the influenza virus. In 1961, David C. Poskanzer and Robert S. Schwab theorized that a viral etiology underpinned the development of Parkinson's disease, and while their prediction was incorrect, a 58-year-old woman with PD reported to them the improvement of her symptoms after receiving amantadine in April 1968. The improvement of the woman's symptoms promoted the first clinical trial to test the effects of amantadine on patients with Parkinson's disease. The clinical trial reported a 66% of improvement in regards to Parkinson's disease symptoms. These results lead to amantadine being approved by the FDA in 1973 as a medication to alleviate Parkinson's disease symptoms [9].

Most of the studies exploring the effect of amantadine were conducted on the adult population, seeing that they are the age group most impacted by extrapyramidal manifestations of Parkinson's disease, while not fully explored in the pediatrics' age group [10]. Furthermore, there is extremely limited data in regards to this topic in the Middle East generally and Saudi Arabia specifically. Taking that into account, the purpose of this case study is to report the outcome of treating a child who developed extrapyramidal symptoms due to organophosphates poisoning with amantadine and to review the literature reporting on its use in treating extrapyramidal manifestations postorganophosphates poisoning.

## 2. Case Report

A 6-year-old boy, with a weight of 20 kg, living in Khamis Mushait (a city 884 kilometers away from the capital Riyadh), was found by his family drowsy and drooling with an insecticide bottle beside him. He was brought immediately to the Emergency Department and arrested upon arrival, and he was eventually revived after 5 minutes of CPR and 5 doses of atropine (0.02 mg/kg/dose). In addition, he received one dose of pralidoxime 50 mg. After that, the patient was intubated and admitted to the pediatric intensive care unit (PICU). He was started on the atropine infusion of 0.2 mg/hour for one hour then decreased to 0.1 mg/hour for another one hour and then infusion was discontinued. After that, he was shifted to divided doses: first, the dose was 0.2 mg every 2 hours for 10 doses, then spaced to every 8 hours with a dose of 0.5 mg for 3 doses and then discontinued. Five days afterward, he was transferred to King Abdullah Specialized Children's Hospital in Riyadh. He was initially admitted to the pediatric intensive care unit (PICU), where he remained for one week. During his PICU stay, he was on antiepileptic medications and morphine. Afterward, he was transferred to the general ward. He then started developing symptoms such as delirium, hyperactivity, akathisia, and aphonia. Initially, gabapentin and chloral hydrate were given as needed in an attempt to alleviate his symptoms. However, there was no improvement, and he started to develop tremors and rigidity with a persistent head tilt. Atropine toxicity was a less likely cause of his symptoms because of the following reasons. First, the dosage of atropine was appropriate for the patient's age and weight and did not exceed the maximum dose, as it was reviewed [11–13]. Second, the half-life of atropine in children older than 2 years is 2.5 ± 1.2 hours, and the symptoms of our patient developed 3 weeks after stopping atropine, which makes atropine overdose less likely [13]. Third, the side effects of atropine most frequently are related to antimuscarinic effects such as tachycardia, constipation, urinary retention, dry mouth, blurred vision because of mydriasis, and fever; however, most of these symptoms were not developed in our patient [14]. Lastly, the patient was severely poisoned, and those patients may have a higher tolerance to atropine and need more dosages, in contrast to patients who are slightly poisoned and may develop atropine toxicity earlier [11]. So, the hypothesis of extrapyramidal symptoms resulting from OP poisoning was made after a thorough literature review was done. Thus, a trial of oral amantadine was started with 50 mg once daily and then increased to 50 mg twice daily. After the start of this attempt, he was assessed daily by the pediatric inpatient team. Throughout his two weeks stay in the general ward, a steady improvement was noted by the family and treating team. Eventually, upon discharge, he was able to walk alone unsupported, with no head tilt, he was also able to play with numbers and animal puzzles correctly, his tremors were almost resolved, speech fluency and clarity were greatly improved, and he no longer experienced agitation. He was followed up postdischarge and showed significant improvement. He continued amantadine for almost 3 months until full resolution of his symptoms and then discontinued it after tapering without any side effects being observed.

## 3. Discussion

Organophosphate poisoning is prevalent in children [15]. Organophosphates cause accumulation of acetylcholine and stimulation of cholinergic receptors, which leads to cholinergic syndrome and muscarinic symptoms [4]. Following OP poisoning, there are four types of neurotoxicity observed. The acute cholinergic crisis, the intermediate phase (generalized muscle weakness), delayed sensorimotor or motor neuropathy and basal ganglia impairment [5].

Basal ganglia impairment, which may manifest as extrapyramidal symptoms, is rarely seen, with only 26 cases reported as of 1978 [5]. Thus, it can often be missed or misdiagnosed, especially in the pediatric population, due to its infrequency. Treatment of the acute crisis is usually atropine, an anticholinergic drug, along with an oxime to reactivate acetylcholinesterase and anticonvulsants for seizure prevention [4]. Moreover, amantadine use has been found beneficial in treating extrapyramidal symptoms following organophosphate poisoning [8].

Hsieh et al. theorized that in order to modulate dopaminergic neurotransmission within the basal ganglia, a crucially low level of acetylcholine is needed, and whenever that threshold is surpassed, the symptoms of basal ganglia impairment may be noted. Moreover, the underdevelopment of the brains of the pediatrics' age group could put them at risk for crossing this threshold relatively easier than their adult peers [16].

In 2005, Shahar et al. reported a similar case with 14-year-old boy that accidentally ingested an eggplant covered with insecticide. Similarly, the boy started experiencing agitation, rigid movement, and confusion after he was stabilized post the initial cholinergic crisis. He was given amantadine 100 mg three times, and his symptoms fully resolved within one week. The dosing was switched to 100 mg twice daily, and he was maintained on that for three months before being weaned off without any side effects or recurrence [5].

All in all, as rare as extrapyramidal symptoms as a consequence of OP are, they should still be considered a possibility in the presence of Parkinson's-like symptoms following organophosphate toxicity. However, atropine toxicity should be ruled out by using the appropriate dose for the patient. Furthermore, the use of amantadine should be explored as a possible option, seeing that it enhances neurotransmission and lacks major adverse effects.

## 4. Conclusion

This case illustrates the promising benefits of using amantadine in treating extrapyramidal manifestations following organophosphate ingestion in children. However, more studies are required and needed to further prove the effects and side effects.
